# Intervention description of pharmacist-facilitated medication reviews in Nordic primary care settings: a scoping review

**DOI:** 10.1080/02813432.2024.2439909

**Published:** 2024-12-27

**Authors:** Karl-Erik Bø, Kjell H. Halvorsen, Elin C. Lehnbom

**Affiliations:** a Department of Pharmacy, Faculty of Health Sciences, UiT The Arctic University of Norway, Tromsø, Norway; b Department of Pharmacy, Uppsala University, Uppsala, Sweden

**Keywords:** Medication review, pharmacists, primary care, Nordic, implementation, TIDieR

## Abstract

**Background:**

Multicomponent interventions are increasingly utilized to tackle the complexity of aging and co-morbid patients. However, descriptions of interventions are generally poor, making it difficult for healthcare providers to implement successful programs.

**Objectives:**

This study aimed to explore the completeness of intervention description of pharmacist-facilitated medication reviews (MRs) in Nordic primary care settings.

**Methods:**

We performed a scoping review of studies reporting on pharmacist-facilitated MRs in Nordic primary care settings. Medline, Embase, CINAHL and Web of Science were searched on 24 January 2024. We used Arksey and O’Mally’s framework for scoping studies and applied an adapted version of the Template for Intervention Description and Replication (TIDieR) checklist to evaluate intervention reporting. The Pharmaceutical Care Network Europe (PCNE) classification of MR levels was used to identify the components of different MRs.

**Results:**

Sixteen studies were included in this scoping review. The studies were conducted in Sweden (*n* = 7), Norway (*n* = 6), Finland (*n* = 2) and Denmark (*n* = 1). Information on the participating pharmacists’ expertise, qualifications and training was fully reported in only two studies. Twelve studies did not provide any information related to intervention cost, dose or duration, making it challenging to estimate the economic impact of the intervention. Only one study made an evaluation of intervention fidelity. Conversely, 15 studies lacked information on this topic which can lead to inaccurate conclusions about the program’s effectiveness.

**Conclusion:**

The studies included in this scoping review do not provide sufficient MR information for intervention replication. We recommend that pharmacy trials use reporting checklists to increase the replicability and transferability of effective interventions.

## Introduction

In the process of adopting, replicating and scaling up evidence-based interventions, it is critical to know the details of how the intervention works [1]. This is particularly true for complex interventions with multiple interacting components. However, intervention studies often emphasize outcomes without adequately detailing the interventions [1,2]. Moreover, the ways in which context challenges the transferability of trial results receive little attention. This lack of explicit reporting hinders the understanding of what contributes to an intervention’s success or failure.

Medication reviews (MRs) can be described as a systematic assessment of a patient’s pharmacotherapy to optimize drug treatment and improve patient outcomes [3]. The endorsement of this intervention is robustly backed by the World Health Organization (WHO) and is considered particularly important in situations of complex pharmacological treatments. Notwithstanding its increasing popularity, ‘medication review’ is an umbrella term used for a wide range of multifaceted interventions [4,5]. The comprehensive scope of this service is illustrated in a recent study where the authors present a list of 28 MR interventions with diverse inputs and activities [6].

Even though the MR rely heavily on listing tools for optimal pharmacotherapy their most prominent feature is the human-to-human interaction. Interprofessional collaboration is a salient aspect of the MR, and the intervention generally includes patient interviews, counseling and follow-ups. In human-based interventions, the ‘evidence’ of a program is built into what practitioners do as they deliver the service. Both un-named and un-measured components can be involved in producing the observed effects [7]. Consequently, the outcomes of MRs are contingent on characteristics of the practice settings and any additional inputs, e.g. efforts and resources provided by research teams or stakeholders, not considered a part of the intervention [8–10].

MRs involving pharmacists have been successfully implemented in both inpatient and outpatient settings in countries such as the US, Canada and the UK. Even though several studies show that these interventions can prevent, identify and solve medication-related problems (MRPs) their ability to improve clinically relevant outcomes is not consistently supported [11–13]. Furthermore, the methodological quality of evidence on MRs is reported to be moderate or low [14–16].

### Pharmacist-facilitated MR in Nordic countries

The Nordic region, including Sweden, Denmark, Finland, Iceland and Norway, has a population of approximately 27.8 million [17]. Notwithstanding these countries’ similar healthcare funding structures, their priorities and services vary. The interest in involving and integrating pharmacists in Nordic primary care settings has only emerged in the last couple of decades [18–22]. Although Sweden, Denmark and Finland have established these services in certain localities, pharmacist-facilitated MRs are still evolving in Nordic countries.

Robust research findings enable healthcare providers and decision-makers to make informed choices about modifying and improving current practices. However, it is difficult to build on, or replicate, research findings without a comprehensive description of an intervention’s separate components and activities. Being unclear about how and why an intervention works can lead to an underestimation of the time, effort and resources required to implement it.

This study aimed to explore the completeness of intervention description of pharmacist-facilitated MRs in Nordic primary care settings.

## Materials and methods

### Identifying the research question

This research aimed to explore the completeness of intervention descriptions of pharmacist-facilitated MRs in Nordic primary care settings. The objectives were to investigate whether researchers provided a rationale for the performing MRs, and if the components of the intervention were described in sufficient detail for replication. Further, we wanted to investigate the reporting of strategies to improve fidelity, and/or assessments of fidelity. The ‘usable innovations’ theoretical framework, developed by the Active Implementation Research Network (AIRN) guided the advancement of our research questions [7]. This framework outlines the initial steps of implementing new programs. The term ‘usable innovations’ refers to new technologies or work methods that are not only proven effective but also clearly defined and operationalized so that they can be implemented consistently and effectively in practice [23]. A usable innovation needs to have a precise explanation of its causal pathway to impact the expected program outcomes, a clear description and operational definitions of the innovation’s essential functions, and a practical assessment of the performance of the practitioners who are using the innovation. Essential functions, also called core components or active ingredients, are the features that make an intervention successful.

**Settings**:

In the context of this study, primary care was limited to settings such as home care/community dwellings, nursing homes and general practices (see Table 1).

**Table 1.** Study characteristics of the included studies.

Author (year)	Country	Study design	Intervention framework provided	PCNE^a^ MR level^35^	Setting
Auvinen (2020) [49]	Finland	RCT^b^	Yes	Advanced	Home care centers
Brandt (2014) [51]	Denmark	Development and test	No	Intermediate	General practice
Davidsson (2011) [43]	Norway	Prospective study	No	Intermediate	Nursing home
Dobszai (2023) [36]	Sweden	Cohort study	Yes	Intermediate	Community-dwellings
Fog (2017) [44]	Norway	Observational before/after	No	Intermediate	Nursing home
Granas (2019) [45]	Norway	Descriptive	Yes	Intermediate	Community-dwellings
Halvorsen (2010) [46]	Norway	Descriptive	No	Intermediate	Nursing home
Halvorsen (2019) [47]	Norway	Descriptive	No	Intermediate	Nursing home
Kari (2018) [50]	Finland	Longitudinal RCT	No	Advanced	Home dwellings
Kersten (2013) [48]	Norway	RCT	No	Intermediate	Nursing home
Lenander (2018) [37]	Sweden	Cross-sectional study	Yes	Intermediate	General practice
Lenander (2014) [38]	Sweden	RCT	No	Advanced	General practice
Lenander (2017) [39]	Sweden	Cross-sectional study	Yes	Intermediate	General practice
Milos (2013) [40]	Sweden	RCT	Yes	Intermediate	General practice
Modig (2016) [41]	Sweden	Descriptive	Yes	Intermediate	General practice
Wickman (2022) [42]	Sweden	Retrospective, descriptive	Yes	Intermediate	Community dwellings

^a^Pharmaceutical Care Network Europe.

^b^ Randomized Controlled Trial.

Intervention reporting in eligible studies of this scoping review was assessed using the Template for Intervention Description and Replication (TIDieR) checklist [24]. The checklist contains minimum recommended items for intervention description related to theory and rationale, essential components of the intervention and context, as well as aspects of modifications and fidelity.

### Study design

We performed a scoping review to investigate the intervention reporting of pharmacist-facilitated MRs in Nordic primary care settings. Scoping reviews are suitable for mapping areas of research literature to identify gaps in the evidence base. Unlike systematic reviews, they seek to explore and describe rather than to produce critically appraised evidence [25].

Our research was guided by Arksey and O’Mally’s methodological framework for scoping studies. The framework describes a five-step approach when scoping literature: (1) Identifying the research question. (2) Identifying relevant studies. (3) Study selection. (4) Charting the data. (5) Collating, summarizing and reporting the results [26].

### Identifying and selecting articles

The search string was developed from three concepts and validated against a test set of pre-defined articles of specific relevance. Text mining tools such as the Yale MeSH analyzer and PubReminer were used to adjust the search string concepts to retrieve the articles in the test set. The translation of the validated Ovid Medline search string for use in Ebsco CINAHL, Ovid Embase and Web of Science, was guided by the Polyglot Search Translator [27]. All databases were searched on 24 January 2024. The electronic search strategy for the Ovid Medline database is provided in Online Appendix 1.

All search results were exported to Endnote desktop for duplication removal. Duplicates were removed using the Endnote de-duplication tool and by manually assessing all the retrieved articles. De-duplicated articles were uploaded to the online software program Rayyan^®^ for screening. All titles and abstracts were screened by one researcher (KEB). Screening questions were developed based on eligibility to enhance clarity during the title and abstract screening process. Articles lacking descriptions of (1) a MR intervention, (2) involvement of pharmacists and (3) a primary care setting were excluded. When in doubt, the articles were included for a second round of screening.

Additional searches were made in Swemed+, a bibliographic database that contains articles from the Nordic countries/Scandinavian journals in medicine and healthcare using the MeSH-term ‘medication review’. This database ceased to be updated in 2020.

Citation searches were performed to locate additional studies, mainly by investigating knowledge synthesis and umbrella reviews on similar topics, e.g. on MRs [4,28–31] and the implementation of pharmacist services in primary care settings [32–34].

### Eligibility criteria

This scoping review included Nordic studies that were peer-reviewed and published from January 2010 to January 2024. Only studies describing pharmacist-facilitated^1^ MRs in primary care settings were included. Studies were excluded if they described MRs conducted in community pharmacies or by community pharmacists; if they were unavailable in English, Swedish, Danish or Norwegian, and if they were conference proceedings or abstracts, posters or comments, letters and opinions.

### Charting the data

Extracted data included author, country, study design, characteristics of the intervention and setting. In addition, the study aim and conclusions were extracted to highlight outcomes and illustrate the importance of describing the intervention (in detail). Extracted data are presented in Tables 1–3.

**Table 2.** Assessment of the MR intervention reporting in each included study.

	1	2	3	4	5a	5b	5c
TIDieR items	Name	Rationale	Materials	Procedures	Expertise	Qualifications	Training
Auvinen 2021 [49]	Reported	Partly reported	Partly reported	Partly reported	Reported	Reported	Partly reported
Brandt 2014 [51]	Reported	Partly reported	Partly reported	Partly reported	Not reported	Not reported	Not reported
Davidsson 2011 [43]	Reported	Partly reported	Reported	Partly reported	Not reported	Not reported	Not reported
Dobszai 2023 [36]	Reported	Partly reported	Partly reported	Partly reported	Partly reported	Partly reported	Partly reported
Fog 2017 [44]	Reported	Partly reported	Partly reported	Partly reported	Not reported	Not reported	Partly reported
Granas 2019 [45]	Reported	Partly reported	Reported	Partly reported	Not reported	Not reported	Partly reported
Halvorsen 2010 [46]	Reported	Partly reported	Reported	Partly reported	Not reported	Not reported	Partly reported
Halvorsen 2019 [47]	Reported	Partly reported	Reported	Reported	Not reported	Not reported	Not reported
Kari 2018 [50]	Reported	Partly reported	Reported	Reported	Reported	Reported	Reported
Kersten 2013 [48]	Reported	Partly reported	Partly reported	Partly reported	Not reported	Not reported	Not reported
Lenander 2018 [37]	Reported	Partly reported	Reported	Reported	Reported	Not reported	Not reported
Lenander 2014 [38]	Reported	Partly reported	Reported	Reported	Reported	Partly reported	Partly reported
Lenander 2017 [39]	Reported	Partly reported	Reported	Partly reported	Reported	Not reported	Not reported
Milos 2013 [40]	Reported	Partly reported	Partly reported	Partly reported	Reported	Not reported	Reported
Modig 2016 [41]	Reported	Partly reported	Partly reported	Partly reported	Not reported	Not reported	Not reported
Wickmann 2022 [42]	Reported	Partly reported	Partly reported	Partly reported	Reported	Reported	Reported
	6	7	8	9	10	11	12
TIDieR items	Delivery mode	Location	Dose	Tailoring	Modifications	Planned fidelity	Actual fidelity
Auvinen 2021 [49]	Reported	Reported	Reported	Not reported	Not reported	Partly reported	Not reported
Brandt 2014 [51]	Reported	Reported	Reported	Not reported	Not reported	Partly reported	Not reported
Davidsson 2011 [43]	Reported	Partly reported	Partly reported	Not reported	Not reported	Not reported	Not reported
Dobszai 2023 [36]	Reported	Partly reported	Partly reported	Not reported	Not reported	Partly reported	Not reported
Fog 2017 [44]	Reported	Partly reported	Partly reported	Not reported	Not reported	Not reported	Not reported
Granas 2019 [45]	Reported	Not reported	Not reported	Not reported	Not reported	Not reported	Not reported
Halvorsen 2010 [46]	Reported	Partly reported	Not reported	Not reported	Not reported	Not reported	Partly reported
Halvorsen 2019 [47]	Reported	Partly reported	Not reported	Not reported	Not reported	Not reported	Not reported
Kari 2018 [50]	Reported	Partly reported	Partly reported	Not reported	Partly reported	Partly reported	Not reported
Kersten 2013 [48]	Reported	Not reported	Partly reported	Not reported	Not reported	Not reported	Not reported
Lenander 2018 [37]	Reported	Partly reported	Partly reported	Not reported	Partly reported	Partly reported	Not reported
Lenander 2014 [38]	Reported	Partly reported	Reported	Not reported	Not reported	Partly reported	Not reported
Lenander 2017 [39]	Reported	Partly reported	Not reported	Not reported	Partly reported	Not reported	Not reported
Milos 2013 [40]	Reported	Partly reported	Partly reported	Not reported	Not reported	Partly reported	Not reported
Modig 2016 [41]	Reported	Not reported	Not reported	Not reported	Not reported	Not reported	Not reported
Wickmann 2022 [42]	Reported	Partly reported	Partly reported	Not reported	Not reported	Partly reported	Not reported

**Table 3.** Description of each study’s rationale for the performing the intervention, aim and conclusion.

Author (year)	TIDieR item 2: Rationale, theory or goal that underpins the intervention or the components of a complex intervention	Aim of study	Conclusion
Auvinen (2020) [49]	Improving medication quality may support the elderly’s functioning. An interprofessional team approach is advantageous when assessing patients with multimorbidity and complex medications.	Testing whether the intervention influenced the number of drugs, drug-drug interactions, risk of drug-induced impairment, medication-related risk load and potential inappropriate medications..	The intervention improved several aspects of medication quality for home care patients.
Brandt (2014) [51]	*An explicit rationale for the MR was not provided. The paper describes a MR practice model.*	To describe and test an MR practice model tailored to the general practice setting.	The model was found to be workable and produced recommendations with high acceptance rates (82%)
Davidsson (2011) [43]	Inappropriate prescribing is associated with increased morbidity, hospitalizations, mortality and cost. Pharmacist-conducted MR shows promising but not conclusive results.	To examine the effect of multidisciplinary, systematic MR on prescribing quality and to evaluate if drug changes were maintained over time.	Multidisciplinary MRs were effective in improving the quality of drug treatment in nursing home patients by reducing the number of drugs and the number of drug-related problems (DRPs).^a^
Dobszai (2023) [36]	The cost of DRPs in elderlies. DRP can be prevented, and the MR can contribute to preventing and reducing DRPs.	To evaluate the MR regarding the clinical relevance of the pharmacists’ recommendations and the implementation of the recommendations by the GP.	The high portion of clinically relevant recommendations from pharmacists emphasizes the importance of MRs to avoid DRPs.
Fog (2017) [44]	Elderlies have an increased risk of adverse drug reactions (ADRs). MRs can improve the quality of drug therapy in nursing homes even though there is a lack of evidence on their effects related to ‘hard’ outcomes.	To describe DRPs identified through multidisciplinary MRs and the interventions that were carried out to resolve them, as well as changes in drug use that followed the MRs.	The MR resulted in overall less drug use, most pronounced for psychotropic drugs and opioids, and in a closer follow-up to optimize the potential benefits of the drug use.
Granas (2019) [45]	A MR should be provided regularly [in patient groups who are more likely to be prescribed potentially inappropriate medication ] to determine adherence, and to monitor effects, adverse effects and interactions.	To describe how interdisciplinary MR may improve pharmacotherapy (…) using MRs and interdisciplinary case conferences. This should contribute to more rational pharmacotherapy (…).	MRs and interdisciplinary case conferences improved pharmacotherapy (…).
Halvorsen (2010) [46]	The prevalence of DRPs is high in nursing homes. Studies have shown that MRs in nursing homes are effective in identifying DRP. Pharmacists’ involvement in MR has been shown to positively impact medication quality.	To describe an innovative team intervention to identify and resolve DRPs in nursing homes.	The intervention was suitable to identify and resolve DRPs in nursing homes.
Halvorsen (2019) [47]	Polypharmacy leads to MRPs. MRPs are associated with hospitalizations, morbidity, mortality and decline in quality of life. MRs have shown promising results in reducing MRPs.	To describe a stepwise, pharmacist-led MR in combination with an interdisciplinary team collaboration to identify, resolve and prevent MRPs in nursing homes (…).	The pharmacist-led MR service was highly successfully piloted with many prevented and solved MRPs.
Kari (2018) [50]	A substantial portion of clinically relevant DRPs identified in MR are discovered by interviewing the patient. The evidence base of MR is not conclusive, but the intervention has been shown to reduce DRPs and increase medication knowledge.	To examine how critical patient involvement is in pharmacist-led MRs and in identifying the most significant clinical DRPs.	Patient involvement is essential when identifying clinical DRPs. Poor therapy control, nonoptimal drug use and intentional or unintentional nonadherence might otherwise be missed.
Kersten (2013) [48]	Polypharmacy and inappropriate prescribing are common in nursing home residents and increase the risk of ADRs and hospitalizations. Clinical pharmacists have been reported to identify a large number of DRPs but the effect of pharmaceutical interventions on relevant patient-oriented outcomes is largely unexplored.	To investigate if reduced anticholinergic drug burden, facilitated by pharmacist interventions, could improve cognitive function in nursing home residents.	Pharmacist-initiated drug changes did not improve cognitive functions in nursing home residents.
Lenander (2018) [37]	Elderly patients (…) risk suffering from DRPs, and a substantial portion of hospital admissions among elderly are due to adverse drug events (ADEs). One way to prevent DRP among elderlies is to carry out MR.	To evaluate the effect of MR in elderly patients in primary care in relation to total drug use and potentially inappropriate drug use, and to describe DRPs.	MRs performed in everyday care are one way of improving drug use among elderlies. The use of potentially inappropriate medications decreased after MR.
Lenander (2014) [38]	Elderly patients (…) risk suffering from DRPs, and a substantial portion of hospital admissions among elderly are due to ADEs. Provision of MR for elderlies with polypharmacy [often: >5 medications] has produced favorable effects.	The primary aim was to assess whether a pharmacist intervention would decrease the number of drugs and the number of DRPs.	The addition of a skilled pharmacist to the primary care team may contribute to reductions in the number of drugs.
Lenander (2017) [39]	Antipsychotic drugs should be used with caution among elderly patients. However, the prescription of antipsychotics in this patient group seems to be high. MR provides a possible strategy to improve the situation.	To assess the effects of MRs on antipsychotic drug use in elderly patients. (…)	MRs appear to offer one useful strategy for reducing excessive use of these drugs.
Milos (2013) [40]	Aging is known to be associated with increased risk of DRPs, higher morbidity and higher numbers of hospital admissions. Multidisciplinary MR has proven to reduce the number of psychotropic drugs in nursing homes.	The primary objective was to assess a structured model of care by studying the impact of pharmacist-led MR on the number of patients using PIMs.	MR involving pharmacists in primary health care appears to be a feasible method to reduce the number of patients with PIMs, thus improving the quality of pharmacotherapy in elderly patients.
Modig (2016) [41]	Aging is known to be associated with increased risk of DRPs, higher morbidity and higher numbers of hospital admissions. A majority of DRPs are preventable. Team-based MR can prevent and solve DRPs.	To evaluate the quality of the clinical pharmacy service to primary care using structured MR, focusing on the clinical significance of recommendations made by the pharmacist.	The high portion of clinically significant recommendations provided by pharmacists when performing team-based MRs suggests that these clinical pharmacy services have the potential to increase prescribing quality.
Wickman (2022) [42]	Elderly patients are prone to polypharmacy which leads to a higher risk of DRPs. MRs can identify and resolve DRPs.	To describe the group of patients considered in need of a pharmacist-led MR, as well as their outcomes regarding DRPs and involved medications.	A majority of the selected patients had at least one DRP. Patients with impaired renal function or polypharmacy may need special attention.

^a^A DRP is defined by PCNE as “an event or circumstance involving drug therapy that actually or potentially interferes with desired health outcomes” [50]. Most studies characterized DRP according to this definition.

This study used the TIDieR checklist, a 12-item checklist developed by an international group of experts, to assess intervention reporting. The 12-item checklist was adapted by specifying the item related to the reporting of interventionists’ expertise, qualifications and training into three separate items [5]. This adaption made it easier to compare the pharmacists’ competencies across studies. Consequently, the 12-item checklist was developed into a 14-item template. The adapted TIDieR checklist is provided in Box 1. As recommended by Hoffman et al. we used the checklist in conjunction with the TIDieR guide [24].

For every included study, each item in the adapted TIDieR checklist was scored ‘reported’, ‘partly reported’ or ‘not reported’. As most TIDieR items comprise several sub-elements it was sometimes difficult to decide whether an item was reported or not. However, as the checklist is considered to contain a minimum of recommended items to describe an intervention, we required every sub-element to be described for an item to be considered ‘reported’. Likewise, items were considered ‘not reported’ when studies provided no information on any sub-element. Incomplete reporting on TIDieR items were scored as ‘partly reported’.

This study addresses pharmacists reviewing patients’ pharmacotherapy regimen, and we relate items 5a–5c in the adapted TIDieR checklist exclusively to this profession.

The Pharmaceutical Care Network Europe (PCNE) classification of MR levels was used to identify the components of different MRs. The PCNE has classified MRs as simple, intermediate and advanced based on their complexity and patient involvement [35]. The most basic type of MR is based solely on medication history (type 1). Intermediate MR type 2a includes medication history and patient interviews, while intermediate MR type 2b includes medication history and clinical data. Advanced MR (type 3) includes medication history, patient interviews and clinical data.

## Results

### Collating, summarizing and reporting of the results

#### Article selection

**Figure 1.**
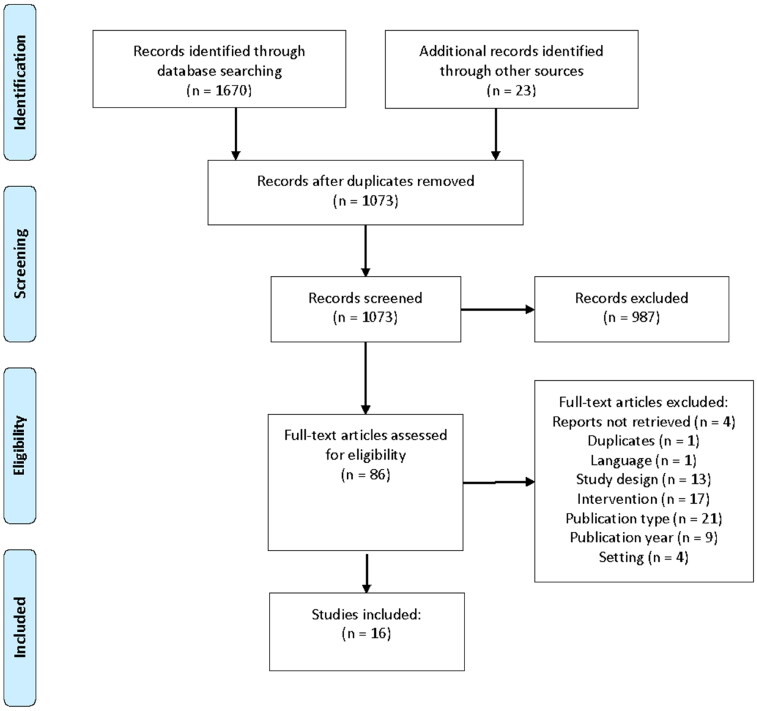
Steps of the study selection process.

A total of 1670 titles were identified, yielding 63 potential studies. Additional records were identified through citation searches and hand searching (*n* = 23). The total number of included studies was 16. The studies were conducted in Sweden [36–42] (*n* = 7), Norway [43–48] (*n* = 6), Finland [49,50] (*n* = 2) and Denmark [51] (*n* = 1). Figure 1 displays the steps in the study selection process. A complete version of the PRISMA flow diagram is provided in Online Appendix 2.

Box 1. Adapted TIDieR checklist.

1. BRIEF NAME: Provide the name or a phrase that describes the intervention.

2. WHY: Describes any rationale, theory or goal of the elements essential to the intervention.

3. WHAT (materials): Describes any physical or informational materials used in the intervention, including those provided to participants or used in intervention delivery or in training of intervention providers.

4. WHAT (procedures): Describes each of the procedures, activities and/or processes used in the intervention, including any enabling or support activities.

5a. WHO (expertise): For each category of intervention provider describe their expertise.

5b. WHO (qualifications): For each category of intervention provider describe their background.

5c. WHO (training): For each category of intervention provider describe specific training given.

6. HOW: Describes the modes of delivery (e.g. face-to-face) and whether it was provided individually or in a group.

7. WHERE: Describes the type(s) of location(s) where the intervention occurred, including any necessary infrastructure or relevant features.

8. WHEN and HOW MUCH: Describe the number of times the intervention was delivered and over what period of time including the number of sessions, their schedule and their duration, intensity or dose.

9. TAILORING: If the intervention was planned to be personalized, titrated or adapted, then describe what, why, when and how.

10. MODIFICATIONS: If the intervention was modified during the course of the study, describe the changes (what, why, when and how).

11. HOW WELL (Planned): Describes strategies used to maintain or improve fidelity (how and by whom)

12. HOW WELL (Actual): Describes the extent to which the intervention was delivered as planned (if adherence or fidelity was assessed)

#### Characteristics of included studies

Study characteristics are provided in Table 1. The interventions described in the included studies were mostly multidisciplinary. However, pharmacists conducted a review of the patient’s pharmacotherapy in all studies. Other team members such as nurses, assistant nurses and physicians were involved in activities such as symptom assessments, patient interviews, counseling and follow-ups.

The included studies in this scoping review tended to remark about the MR as a uniform service. However, referring to ‘medication review’ imprecisely describes the multiple components of the intervention. The description of each study’s MR level according to PCNE classification was not explicitly stated in the included studies. Consequently, an assessment of PCNE MR level was made based on the information provided. All studies reported having access to clinical data and the patient’s medication history. However, only three studies reported including patient interviews as part of the intervention, i.e. performing advanced type 3 MRs (see  Table 1) [38,49,50]. Studies that did not report conducting patient interviews were categorized as intermediate type 2b MRs.

Table 2 provides a representation of the completeness of intervention descriptions in the included studies of this scoping review. The items were scored as reported (green), partly reported (yellow) and not reported (red). The complete TIDieR checklist is provided in Online Appendix 3.

#### Description of the intervention and its materials and procedures (TIDieR items 1–4)

A brief name and description were found for all interventions. Even though most studies referred to the intervention simply as ‘(clinical) medication reviews’ [36,38,39,41–47,50,51] or ‘drug review’ [48], some studies referred to conceptual frameworks in naming their intervention, e.g. the Finnish Interprofessional Medication Assessment model (FIMA) [49], the Lund Integrated Medicine Management model (LIMM) [37,40] and the Integrated Medicine Management (IMM) model [45].

Item 2 of the TIDieR checklist covers the rationale, theory and goal of the intervention. A program theory explains how a program contributes to both intermediate results and the observed outcomes [52]. Furthermore, it can help identify which elements of an intervention are essential and which are optional or less important. Table 3 provides an overview of the background (theory), study aim (goal) and study conclusions.

In most studies, the intervention logic assumes that MRPs are prevalent and can be identified through an assessment of the patient’s pharmacotherapy (see Table 3). International evidence of similar interventions, and/or outcomes produced by similar interventions in healthcare settings were used to underpin this rationale. Several studies emphasized that the evidence supporting MRs to provide clinically relevant patient outcomes is inconclusive [36,43,44,48–50].

Despite MRs’ multifaceted and interprofessional nature, most studies did not assess the importance of the intervention’s separate components. Only one study specified an intervention component they considered to be essential for producing the expected outcomes [50]. Another study discussed the challenge of determining the contribution of separate intervention components to the observed outcomes [38].

Most studies reported, or partially reported, intervention materials. Overall, the authors provided comprehensive descriptions of intervention materials such as criteria lists, assessment tools, databases and access to medical records and clinical data. However, none of the studies described any material used in the training of pharmacists conducting the pharmacotherapy reviews.

All studies provided some information on the procedures, activities and processes used in the intervention. Several studies referred to frameworks to describe the components of their intervention, e.g. FIMA [49], LIMM [36,37,39–42] and IMM [45]. Non-standardized intervention descriptions outlined its sequence of steps and defined each team member’s role.

#### Description of the pharmacist’s expertise, qualifications and training (adapted items 5a–5c)

The interventions described in the included studies were interprofessional, comprising efforts from nurses, assistant nurses, physicians and pharmacists. However, only pharmacists performed the assessment of each patient’s pharmacotherapy, aiming to optimize treatments and improve patient outcomes.

The level of detail reported on pharmacists’ expertise, qualifications and training was consistently low across most studies. Furthermore, descriptions of these items were brief, typically comprising a couple of sentences. Only five studies reported, or partly reported, information on all three items [36,38,42,49,50]. One study provided information on two items [40]. Five studies reported, or partly reported, information on only one item [37,39,44–46], and five studies did not provide any information on any item [41,43,47,48,51].

#### Description of the intervention’s mode of delivery, setting and dose (TIDieR items 6–8)

Information on the intervention mode of delivery (item 6) was provided indirectly rather than explicitly stated. As the MRs in the included studies were interprofessional, there were different modes of delivery for the separate components of the intervention. Face-to-face activities with the patient, such as symptom assessments, and patient interviewing were performed by either nurses or pharmacists. Pharmacists independently conducted the pharmacotherapy reviews, likely without the involvement of other healthcare personnel. Inter­professional case conferences following patient contact, and the reviewing of patients’ pharmacotherapy, were reported to be face-to-face interactions.

Intervention setting was reported across all studies, e.g. ‘nursing home’ or ‘general practice’. However, the included studies did not elaborate on location details, or any specific facilities or infrastructure required to perform the intervention (item 7). Descriptions of ‘when and how much’ (item 8) related mostly to the research study period, e.g. ‘*the study was performed during a 15-month period*’.

In general, descriptions concerning intervention frequency, intensity and dose were not reported. Information on intervention duration was reported in only four studies [38,44,49,51]. Other studies provided indirect information on the intensity and dose of the intervention, e.g. ‘*pharmacists conducting the MRs may have been extra thorough in their work since they knew they were part of a study*’ [36,42]. The authors considered that this increased ‘dose’ of the intervention possibly affected study outcomes. Other authors indicated that the intervention was part of everyday practice and provided without additional resources [37,39]. Only one study in this scoping review explicitly reported on the cost of the intervention [38]. However, Kari et al. reflected on the importance of a cost-effectiveness assessment of MRs ‘*as resources in health care are scarce and must be allocated efficiently*’ [50]. All over, 12 studies did not provide any information related to intervention cost, dose or duration, making it challenging to estimate the economic impact of the intervention.

#### Descriptions of intervention modifications and fidelity (TIDieR items 9–12)

According to the TIDieR guide, tailoring relates to whether the intervention was planned to be provided identically to every patient. As specified in the TIDieR checklist, this concerns the reporting on intentional flexibility, i.e. the possibility of customizing the intervention to obtain the appropriate dose for each patient. None of the included studies provided any information on planned intervention flexibility (item 9).

Unforeseen events might happen during a research study making it necessary to alter components of the intervention. Modifications of the intervention (item 10) relate to any changes or adjustments made at the study level. Only three studies described modifications made during the study period [37,39,50].

Items 11 and 12 address intervention fidelity. This involves describing strategies to maintain or enhance intervention delivery, and assessing whether the intervention was delivered as planned. Fidelity reporting extends beyond providing a receipt of the intervention to describe ‘how well’ the intervention was received or delivered. Eight studies reported some strategies to maintain intervention fidelity [36–38,40,42,49–51]. These strategies involved descriptions of the pharmacists’ competencies and training [40,42,49,50], as well as the intervention dose [38,51]. However, intervention fidelity was poorly reported across all studies.

## Discussion

Our investigation of the reporting of pharmacist-facilitated MRs in Nordic primary care settings indicates that they lack intervention clarity. Missing intervention information related most frequently to pharmacists’ expertise, qualifications and training (items 5a–5c), descriptions of intervention frequency, intensity and dose (item 8), and intervention flexibility and fidelity (items 9–12).

### What is the rationale for providing MRs in Nordic outpatient settings?

A common approach to quality improvement in healthcare is to do what others do [53]. However, attempting to replicate multicomponent interventions with a previous evidence base requires an understanding of the essential functions of the original intervention and the interplay between the original intervention and its context [54]. Even though national or regional guidelines in Nordic countries recommend performing MRs, adaption and tailoring to local conditions are always necessary [55]. Moreover, recognizing the theoretical assumptions that underpin the intervention is pivotal [56,57].

All the included studies in this scoping review performed an impact evaluation of a new intervention. When performing this kind of research, the overarching objective is to advance knowledge and improve outcomes. The results from experimental studies often inform healthcare practitioners and decision-makers about new and effective programs. Indeed, the included studies in this scoping review advocate MRs as a service to optimize medications and improve patient safety. However, MRs mostly impact softer outcomes, or ‘*measurable variables with an indirect or unestablished connection to*’ [58] target outcomes such as adverse events, quality of life or mortality [59–63]. This is partly reflected in the terminology of some of MR end results, such as *potentially* inappropriate medications (PIMs) or *possibly* omitted medications.

An assessment of the cost-benefit of MRs is outside the scope of this study. However, the progressively aging and co-morbid population drives health expenditures making it increasingly important to spend money wisely and to implement services with a solid evidence base. Decision-makers are increasingly expected to consider complex interventions and successful implementation hinges on clear and complete intervention descriptions. An important economic aspect to assess in implementation is the affordability of healthcare services, i.e. whether an intervention can be afforded regardless of its effectiveness [64]. The absence of cost data in MR trials might hinder decision-makers from prioritizing healthcare investments knowledgeably.

### Descriptions of pharmacists’ expertise, background and specific training

The characteristics of the intervention provider can impact the outcomes of the intervention. Important aspect to address includes specific skills, expertise and experience required by the providers. Pharmacists have different educations, levels of expertise and abilities. Even the personal traits of pharmacists, such as insecurity and fear of new responsibilities, are known to influence how they perform in pharmacy practice [65]. Consequently, terms such as ‘pharmacists’ or ‘clinical pharmacists’ do not sufficiently describe the competencies of the intervention provider. Additional information such as ‘vast experience’ does not necessarily make their expertise more precise.

The training of participating health personnel is a critical component of successful program implementation. Providing tuition and coaching is important to ensure competence, enhance confidence and maintain intervention fidelity. Educating stakeholders on the new program can help facilitate adaptation and improve the development of strategies for implementation.

The lack of reporting on these TIDieR items are in line with the results of research investigating the reporting of similar interventions. de Barra et al.’s systematic review of pharmacy interventions investigated pharmacist services implemented in outpatient settings, e.g. MRs and medication counseling. They found that trial reports insufficiently reported on the experience, qualifications and training of pharmacists [5].

### Planned intervention versus actual intervention

Intervention fidelity is a multidimensional construct on both quantitative and qualitative components of a treatment or program. Generally, intervention fidelity refers to the methodological strategies used to monitor and enhance the reliability and validity of interventions [66] Poor fidelity makes it difficult to attribute outcomes directly to the intervention, leading to inaccurate conclusions regarding its effectiveness. This may result in the implementation and continuation of ineffective practices or the premature dismissal of potentially beneficial ones.

Descriptions of strategies to improve and assess intervention fidelity were poorly reported across all trials. This is unfortunate since all studies concluded that their interventions successfully produced the intended outcomes (see Table 3) Notwithstanding the limited possibility of providing necessary intervention details in the primary paper, authors should describe the activities and state where the information is located [24].

Poor fidelity reporting is not a new phenomenon, and our results are in line with the findings from similar studies. A 2018 systematic review deemed pharmacist interventions in asthma management as unimplementable due to inadequate intervention fidelity [67]. A scoping study evaluating the implementation of multidisciplinary practices to improve pharmacotherapy, e.g. MR, found that none of the included studies evaluated fidelity [68]. Even though intervention fidelity and implementation fidelity focus on different processes they are closely related. It is difficult to achieve high implementation fidelity with low intervention fidelity.

Naming the interventions according to standardized terminologies such as FIMA of LIMM made it easier to conceptualize the scope of MR. However, studies using the ‘Pharmacotherapeutical Symptom Evaluation, 20 questions’ (PHASE-20) tool as part of their MR reported inconsistently on its application [36,37,41,42]. This variability underscores the need for explicit reporting on the administration of standardized tools, as the mode of collection can influence the quality and nature of the data obtained. Furthermore, using standardized terminologies such as the PCNE classification can make it easier to identify the foundation of the intervention, e.g. patient interview, medication history and/or clinical data. Reporting frameworks specifically designed for pharmacist interventions exist, e.g. Descriptive Elements of Pharmacist Intervention Characterization Tool (DEPICT2) [69].

### Limitations

This scoping review has several limitations. MR is an umbrella term encompassing a plethora of multifaceted and interprofessional interventions. The complexity of the MR intervention makes it likely that our search failed to retrieve relevant information on the topic. Furthermore, in some studies, it was difficult to conceptualize the pharmacist’s responsibilities within an interprofessional practice. Studies failing to explicitly mention the pharmacists’ role in the intervention were excluded. Furthermore, using the TIDieR checklist to evaluate intervention reporting proved challenging, as the details provided did not always clearly align with the checklist items. Similar problems have been reported in other studies [5,70].

## Conclusion

This study provides an overview of how Nordic studies describe pharmacist-facilitated MRs in primary care settings. In general, the trials we reviewed did not make any fidelity assessments, nor did they provide information on the dose and cost of the intervention. Whether each trial’s reporting is sufficient for other settings to replicate its positive outcomes might depend on the objectives of different stakeholders. However, insufficient information about the intervention may lead to misinterpretation of cause and effect.

Decision-makers are increasingly expected to consider complex interventions and successful implementation hinges on clear and complete intervention descriptions. Furthermore, understanding the context of an intervention is key to successful delivery. Consequently, we recommend that pharmacy trials use reporting checklists, e.g. the TIDieR, to increase the replicability of pharmacist interventions such as the MR.

## Supplementary Material

2024 Appendix 3 Adopted TIDieR checklist.docx

2024 Appendix 2 PRISMA Flow diagram.docx

2024 Appendix 1 Ovid Medline search string.docx

## Notes

1 Studies were excluded if the MR intervention was conducted without pharmacists.
