# Supplementary material for: Intervention description of pharmacist-facilitated medication reviews in Nordic primary care settings: a scoping review
Source: Scand J Prim Health Care. 2024 Dec 27;43(1):241–53. doi: 10.1080/02813432.2024.2439909 (PMC11834788; doi:10.1080/02813432.2024.2439909)
Supplement: 2024 Appendix 3 Adopted TIDieR checklist.docx [file IPRI_A_2439909_SM4717.docx]

## Appendix 3: adapted Template for Intervention Description and Replication checklist^1^.

Reviewers- use “?” if information about the element is not reported/not sufficiently reported.

^1^ Hoffmann T, Glasziou P, Boutron I, Milne R, Perera R, Moher D, Altman D, Barbour V, Macdonald H, Johnston M, Lamb S, Dixon-Woods M, McCulloch P, Wyatt J, Chan A, Michie S. Better reporting of interventions: template for intervention description and replication (TIDieR) checklist and guide. BMJ. 2014;348:g1687.

Reviewers- use “?” if information about the element is not reported/not sufficiently reported.
