# Supplementary material for: Intervention description of pharmacist-facilitated medication reviews in Nordic primary care settings: a scoping review
Source: Scand J Prim Health Care. 2024 Dec 27;43(1):241–53. doi: 10.1080/02813432.2024.2439909 (PMC11834788; doi:10.1080/02813432.2024.2439909)
Supplement: 2024 Appendix 2 PRISMA Flow diagram.docx [file IPRI_A_2439909_SM4716.docx]

**Identification of studies via other methods**

**Identification of studies via databases and registers**

Records identified from:

Websites (n =1)

Citation searching (n = 22)

Records removed *before screening*:

Duplicate records removed (n = 620)

Records identified from*:

Databases (n = 1670)

Ovid Medline (n=395)

Ovid Embase (n=568)

Ebsco Cinahl (n=206)

Web of Science (n=501)

**Identification**

Records screened

(n = 1050 )

Records excluded**

(n = 987)

Reports not retrieved

(n = 2)

Reports sought for retrieval

(n = 23)

Reports sought for retrieval

(n = 63 )

Reports not retrieved

(n = 2)

**Screening**

Reports assessed for eligibility

(n = 21)

Reports excluded:

Publication year (n = 9)

Setting (n = 4)

Intervention (n = 6)

Study design (n = 1)

Publication type (n = 1)

Reports assessed for eligibility

(n = 61)

Reports excluded:

Duplicates (n = 1)

Interventionist (n = 8)

Intervention (n = 3)

Publication type (n = 20)

Study design (n= 12)

Language (n = 1)

Studies included in review

(n = 16)

Reports of included studies

(n = 0)

**Included**

*Consider, if feasible to do so, reporting the number of records identified from each database or register searched (rather than the total number across all databases/registers).

**If automation tools were used, indicate how many records were excluded by a human and how many were excluded by automation tools.

*From:*  Page MJ, McKenzie JE, Bossuyt PM, Boutron I, Hoffmann TC, Mulrow CD, et al. The PRISMA 2020 statement: an updated guideline for reporting systematic reviews. BMJ 2021;372:n71. doi: 10.1136/bmj.n71. For more information, visit: <http://www.prisma-statement.org/>
