# Supplementary material for: Intervention description of pharmacist-facilitated medication reviews in Nordic primary care settings: a scoping review
Source: Scand J Prim Health Care. 2024 Dec 27;43(1):241–53. doi: 10.1080/02813432.2024.2439909 (PMC11834788; doi:10.1080/02813432.2024.2439909)
Supplement: 2024 Appendix 1 Ovid Medline search string.docx [file IPRI_A_2439909_SM4715.docx]

**Ovid MEDLINE(R) and Epub Ahead of Print, In-Process, In-Data-Review & Other Non-Indexed Citations, Daily and Versions 1946 to January 24, 2024**

| **#** | **Searches** | **Results from jan 24 2024** |
| --- | --- | --- |
| 1 | "medication review"/ | 145 |
| 2 | "Drug Utilization Review"/ | 3881 |
| 3 | Medication Therapy Management/ | 2811 |
| 4 | Medication Reconciliation/ | 1577 |
| 5 | ((medication* or medicine* or drug*) adj3 (manag* or review* or reconcil* or concord* or assess*)).tw,kf. | 75990 |
| 6 | LIMM.tw,kf. | 13 |
| 7 | Drug utili#ation review.tw,kf. | 416 |
| 8 | (Cognitive adj3 servic*).tw,kf. | 547 |
| 9 | exp Aged/ | 3480165 |
| 10 | primary health care/ or patient-centered care/ | 114363 |
| 11 | Home Care Services/ | 36658 |
| 12 | exp Residential facilities/ | 58927 |
| 13 | community health nursing/ or home health nursing/ | 20143 |
| 14 | Family Practice/ | 67380 |
| 15 | nursing homes.tw,kf. | 20568 |
| 16 | home care.tw,kf. | 22631 |
| 17 | patient-centered care.tw,kf. | 8312 |
| 18 | primary care.tw,kf. | 149962 |
| 19 | Domiciliary care.tw,kf. | 360 |
| 20 | Elderl*.tw,kf. | 302052 |
| 21 | exp "Scandinavian and Nordic Countries"/ | 222814 |
| 22 | Scandinavia*.tw,kf. | 10451 |
| 23 | Skandinavia*.tw,kf. | 1 |
| 24 | Nordic.tw,kf. | 9310 |
| 25 | Nordisk.tw,kf. | 1018 |
| 26 | (Norway or Sweden or Denmark or Finland or Iceland).tw,kf. | 150400 |
| 27 | (Norwegian or Swedish or Danish or Finnish or Icelandic).tw,kf. | 127480 |
| 28 | 1 or 2 or 3 or 4 or 5 or 6 or 7 or 8 | 81433 |
| 29 | 9 or 10 or 11 or 12 or 13 or 14 or 15 or 16 or 17 or 18 or 19 or 20 | 3843882 |
| 30 | 21 or 22 or 23 or 24 or 25 or 26 or 27 | 333745 |
| 31 | 28 and 29 and 30 | 549 |
| 32 | limit 31 to yr="2010 -Current" | 395 |
